# The Ecological and Geographic Context of Morphological and Genetic Divergence in an Understorey-Dwelling Bird

**DOI:** 10.1371/journal.pone.0085903

**Published:** 2014-02-07

**Authors:** Ângela M. Ribeiro, Penn Lloyd, W. Richard J. Dean, Mark Brown, Rauri C. K. Bowie

**Affiliations:** 1 Percy FitzPatrick Institute, DST/NRF Centre of Excellence, University of Cape Town, Cape Town, South Africa; 2 Department of Integrative Biology and Museum of Vertebrate Zoology, University of California Berkeley, Berkeley, California, United States of America; 3 School of Life Sciences, University of KwaZulu-Natal, Pietermaritzburg, South Africa; Institute of Evolutionary Biology (CSIC-UPF), Spain

## Abstract

Advances in understanding the process of species formation require an integrated perspective that includes the evaluation of spatial, ecological and genetic components. One approach is to focus on multiple stages of divergence within the same species. Species that comprise phenotypically different populations segregated in apparently distinct habitats, in which range is presently continuous but was putatively geographically isolated provide an interesting system to study the mechanisms of population divergence. Here, we attempt to elucidate the role of ecology and geography in explaining observed morphological and genetic variation in an understorey-dwelling bird endemic to southeastern Africa, where two subspecies are recognized according to phenotype and habitat affinity. We carried out a range-wide analysis of climatic requirements, morphological and genetic variation across southeast Africa to test the hypothesis that the extent of gene flow among populations of the brown scrub-robin are influenced by their distinct climatic niches. We recovered two distinct trends depending on whether our analyses were hierarchically structured at the subspecies or at the within subspecies level. Between subspecies we found pronounced morphological differentiation associated with strong reproductive isolation (no gene flow) between populations occupying divergent climatic niches characterized by changes in the temperature of the warmest and wettest month. In contrast, within subspecies, we recovered continuous morphological variation with extensive gene flow among populations inhabiting the temperate and sub-tropical forests of southern Africa, despite divergence along the climate axis that is mainly determined by minimum temperature and precipitation of the coldest months. Our results highlight the role of niche divergence as a diversifying force that can promote reproductive isolation in vertebrates.

## Introduction

Understanding the factors affecting the process of speciation has been a topic of enthusiastic debate in evolutionary biology for decades (e.g., [1], [2], [3], [4]). Although most evolutionary biologists agree that speciation can be conceptualized as a continuum, i.e. a gradual accumulation of genetic differences and reproductive isolation, the nature of the process is still contentious. For instance, the relative importance of the geographical and ecological components still prompts intensive debate [5], [6], [7].

Recently, an alternate conceptual approach to the study of speciation has been proposed. It incorporates micro-evolutionary concepts into the framework to address how geography, ecology and genetics interact to restrict gene flow between populations [8], [9], [10], [11]. This approach views geographical isolation as a process resulting from the failure of individuals to adapt to the novel environmental conditions that disrupt a once continuous range [12]. As natural selection “pushes” individuals far from the area where fitness is low, migration between populations is reduced and consequently gene flow is diminished. Depending on the extent to which local conditions affect the demography of particular phenotypes (i.e. natural selection), populations may diverge adaptively and thereby possibly attain reproductive isolation, or the process of divergence can be reversed if migration and gene flow resume (e.g., [13]).

**Figure 1:**
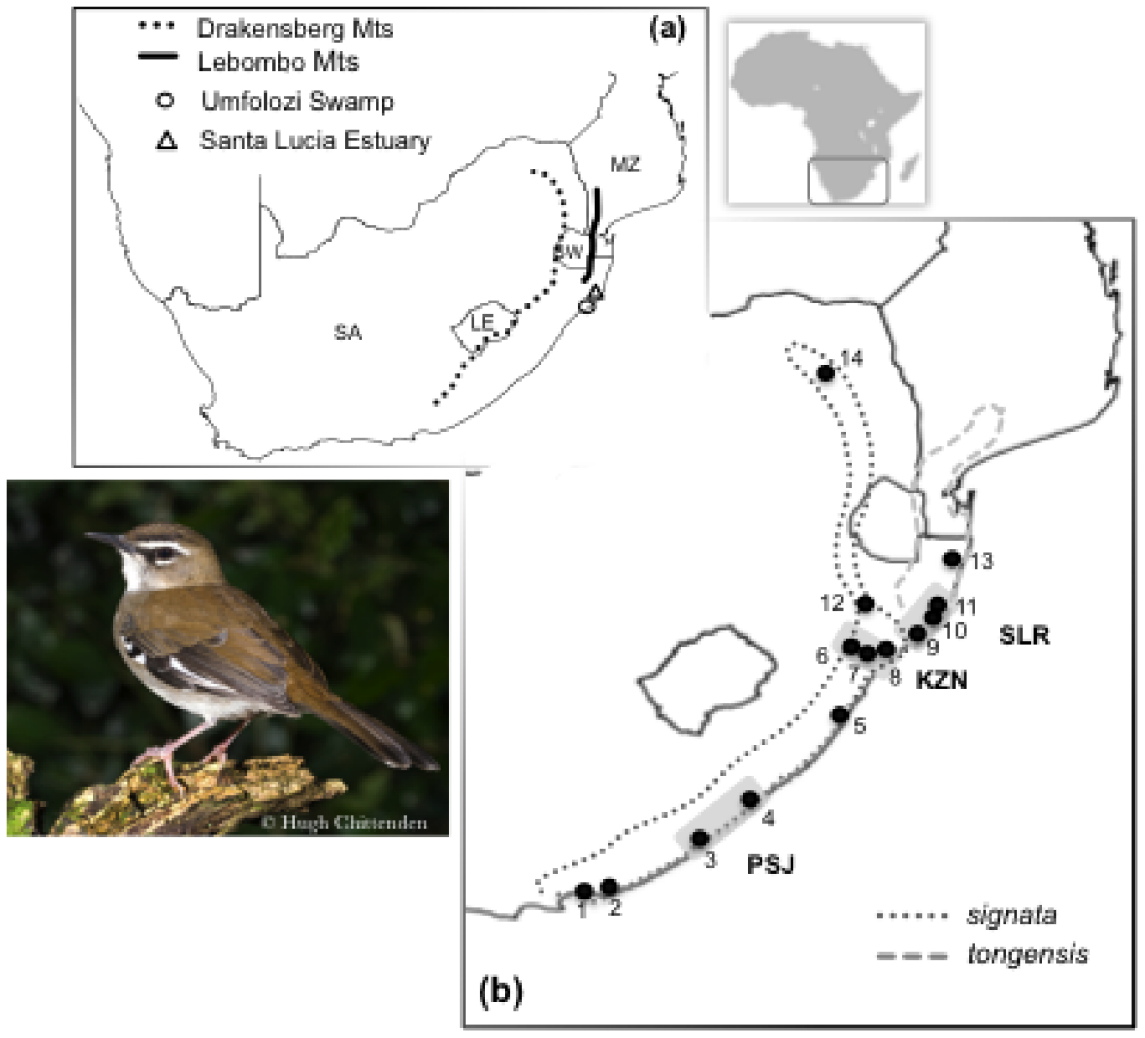
Map of the study area. (a) Depiction of the primary geographical features along the southeastern coast of Africa and position of the mid-winter surface isotherm (18°C). SA - South Africa, LE- Lesotho, SW - Swaziland, MZ - Mozambique. (b) Geographical distribution of the brown scrub-robin, *Cercotrichas signata,* with the range of the two subspecies (*C. s. signata* and *C. s. tongensis*) depicted. The points mapped and numbered correspond to sites from where ecological, morphological and genetic data were gathered. The three main forest regions used in the analyses are annotated as PSJ: Port St. Johns coastal scarp forest in the Eastern Cape; KZN: coastal and interior scarp forest in KwaZulu-Natal; and SLR: Indian Ocean coastal forest in the vicinity of St. Lucia Estuary in northern KwaZulu-Natal, South Africa. The inset depicts the location of our study system in the African continent. Brown scrub-robin photography by Hugh Chittenden.

Species comprising phenotypically different populations occupying distinct habitats, despite an overall apparently contiguous distribution, provide intriguing systems in which to study the underlying population-level mechanisms that promote divergence. The elusive brown scrub-robin *Cercotrichas signata* that inhabits forest understorey habitats in southern Africa is one such species. It comprises two subspecies that occupy adjoining ranges: i) the dark olive-brown *C. s. signata* spans the temperate and subtropical forests of coastal Pondoland and the highlands of Swaziland, Mpumalanga and Limpopo; and ii) the paler, smaller and shorter-billed *C. s. tongensis* is restricted to the lowland tropical forests of the Maputaland region [14] (Figure 1). The boundary between the ranges of the two subspecies is centered on the coastal plains between St. Lucia Estuary and the Umfolozi Swamp [14] and coincides with the region where the 18°C mid-winter isotherm (mean temperature) crosses southern Africa. The habitat of this bird was dramatically affected by the dry and cold conditions that dominated southern Africa during the Last Glacial Maximum [15]. The two major forest regions that persisted in the coastal scarp near Port St. Johns [16] and along the escarpment of northern KwaZulu-Natal [15] are thought to have acted as refugia from which forest expanded with the amelioration of climate following glacial retreat [15], [17]. Further, the coastal tropical forest with East African affinities expanded as far south as the Umfolozi Swamp region with climatic amelioration during the Holocene [18]. The diversity of climatic zones created by historical climate changes over the peculiar topography has been proposed as an important mechanistic trigger of divergence and speciation in southeastern Africa (e.g., [16], [17], [19]), such that this area is recognized as a biodiversity hotspot, the Maputaland-Pondoland-Albany Biodiversity Hotspot [20]. The system above provides an interesting opportunity to examine whether variation in climate may have determined patterns of intraspecific geographical variation in phenotypic traits and molecular genetic markers and hence contributed to population divergence and ultimately speciation.

In this study, we initially modelled climatic niches and quantified the differences to then test for any relationship with morphological divergence and whether increased differences in climatic niche leads to reduced gene flow. Niche differences can be estimated using GIS data and spatial statistics [21], [22], [23] and consequently used to predict the degree of gene flow across the landscape. If populations of the brown scrub-robin diverged along the climatic axis of the niche and this ecological setting is maintaining their separation, we expect to find an inverse relationship between niche divergence and gene flow. We quantified morphological divergence with the rationale that body mass and body size have functional consequences for multiple life-history features, and are therefore subject to selective pressures [24]. This is particularly pertinent because eco-morphological studies have demonstrated that geographical variation in body size, particularly among birds, results from the interaction of species-specific physiology with the environment and resource availability [25]. If climate has been responsible for driving and maintaining morphological divergence, we expected morphological differences to co-vary with measures of niche divergence estimated in the previous step of our analytical framework.

We demonstrate that our analytic framework provides a means to understand where along the divergence continuum (see [26], [27]) brown scrub-robin subspecies lie. Additionally, this framework provides insight into the underlying mechanisms that have resulted in the present stage of divergence and the factors that may either be hindering completion of the process (reproductive isolation) or lead to a reversal of the process of divergence (introgression).

## Methods

### Ethics Statement

The South African National Parks and the South African Provincial Departments granted research and collecting permits to capture birds using mist-nets and flap traps. The capture and sampling were conducted with approval from the Animal Use committees of the University of California Berkeley, the University of Cape Town, and the South African bird-banding scheme (SAFRING).

### Ecological and Geographical Context

Characterizing geographic distributions and testing whether climatic factors influence subspecies ranges serve as a proxy to assess the ecological similarities or differences among subspecies.

In order to assess for equivalency, i.e. overlap, in the climatic niches of *signata* and *tongensis*, we constructed environmental niche models using MaxEnt v.3.3.3. [28], [29]. The geographically explicit environmental niche models were generated based on eight independent bioclimatic variables extracted for each of the geo-referenced occurrence records of brown scrub-robin. From the 19 climatic variables available from the WorldClim database at a resolution of 30 arc-seconds [30], we retained only those showing Pearson's correlation coefficients *r* <0.80. The presence-only point locality data used in these models were primarily obtained from our field expeditions and supplemented with museum specimen records of adult birds as well as ringing records kindly provided by Craig Symes. We modelled the climatic niche supporting the occurrence of the species (36 localities) and also the two subspecies independently (28 sites for *signata* and 8 sites for *tongensis*; Table SI). The model performance was assessed by estimating the “area under the receiver operating curve” – AUC. The predicted probability of occurrence obtained was spatially projected using ArcMap 9.3 [30]. The geographical extent of overlap of the climatic niche of the two subspecies, and thus potential co-occurrence, was determined by converting the cumulative prediction obtained with MaxEnt into binary prediction models, i.e. presence versus absence, which were then summed to produce a map of niche overlap. All the routines involving GIS were performed in ArcMap 9.3 [30]. Converting model predictions into binary format relies on a threshold that reflects when an individual is present in a given pixel. Since the brown scrub-robin is territorial year-round and seasonal movements have not been recorded [14], we made the simplifying assumption that occurrence data reflects a resident bird.

Assessing niche overlap as the graphical output of the niche models projected onto geographical space has an inherent problem: spatial autocorrelation in environmental variables. To account for the possible influence of autocorrelation in the GIS data used, while testing for niche equivalency, i.e. whether there is a difference in the climatic niche occupied by the two subspecies, we implemented a multivariate approach as follows. We used a principal components analysis (PCA) to reduce the 19 climatic variables mentioned in the previous section into a small and uncorrelated set of axes. Differentiation in climatic niche was then quantified as the difference of means along each orthogonal PC axis: *Di observed* (*signata* – *tongensis*). The null distribution for *Di* was generated by randomly reshuffling the data. All occurrences were pooled regardless of their origin, randomly allocated into two groups (subspecies) with the same number of samples as the original data set and *Di null* (*signata* – *tongensis*) estimated. This procedure was repeated 9999 times to generate the null distribution. The observed test statistic (*Di observed*) was then compared to the distribution of the simulated values (D*i null)* to examine whether the observed value was significantly different from zero. The estimated probability of obtaining a result that exceeded the observed value under the null hypotheses was estimated as *p* = [(number *Di null*≥*Di observed*)/total number randomizations]. The same procedure was implemented to estimate niche differentiation between i) contiguous populations across the subspecies boundary: populations from KwaZulu-Natal scarp (KZN) *versus* populations from the St. Lucia Region (SLR); and ii) populations within subspecies *signata*: populations from KwaZulu-Natal scarp (KZN) *versus* populations from Port St. Johns coastal scarp (PSJ). KZN and PSJ are thought to have supported forest remnants during the Last Glacial Maximum (Eeley et al. 1999; Hughes et al. 2005). All statistical analyses were performed using R 3.0 [31]; the script is provided as supplementary material.

### Morphological Divergence

Morphological data were collected from 81 individuals from 14 localities across the range of the species: 55 *C. c. signata* from ten localities and 26 *C. c. tongensis* from four localities (Table S2). As samples sizes from four locations within the range of *C. c. signata* were small, we used geographical proximity and prior knowledge of occupied forest type to reduce the four locations into two. Therefore, morphological analyses were performed with individuals grouped into 12 sites (n_per site_>3). Tarsus-length was measured with a digital calliper to an accuracy of 0.1 mm, wing-length (from the carpal joint to the tip of the longest primary feather) was measured with a wing-rule to an accuracy of 0.5 mm, and body mass was measured with a 50 g Pesola spring balance to an accuracy of 0.5 g.

Body mass (g), wing-length (mm) and tarsus-length (mm) of adult birds (n = 81: 29 females and 52 males) were used to quantify morphological variation across the entire range of the species. The sex of the birds was determined using a PCR-based assay [32]. Prior to analyses, morphological data were log_10_ transformed to meet assumptions of normality. We used the residual values from the regression of log-transformed wing on tarsus as a proxy of structural body size. Exploratory scatterplots indicated morphological variation within the range of the species and between sexes (Figure S1). Therefore, we performed a factorial analysis (univariate ANOVA) to test whether morphology was affected by geography. Because we found sexual dimorphism (see results), we also tested for the effect of geography on sexual dimorphism, which in our analyses is represented by the interaction term, geography*sex. The effect of climate on the extent of morphological variation was assessed using a regression approach. We used PC1 and PC2 on the climatic data as the explanatory variables, because these two components together accounted for as much as 78.42% of the variation observed in the climatic data. In addition to the previous linear models, we also tested for a non-linear relationship between morphology and climate, by including a quadratic term in the model. The rationale behind this was that a subtle change in the climatic conditions could have a dramatic effect on phenotype. We compare the fit of the alternative models by estimating the Akaike Information Criterion corrected for the effect of small sample sizes and number of parameters (AICc; [33]) between each model and best-fitting model (Delta-AICc). We also estimated Akaike weights: the proportion of support received by a model relative to the total support of all models [33]. All statistical analyses were performed using R 3.0 [31].

### Genetic Variation

#### DNA amplification

Blood or muscle samples collected in the field were used to quantify genetic variation. We extracted genomic DNA from 74 blood/tissue samples using DNeasy kits (Qiagen, Valencia, USA). We sequenced two mitochondrial protein-coding genes (ATP6 and ATP8; [34]) and six nuclear introns: Gapdh-intron11 (*Gallus gallus* chromosome 1, [35]), TGFb2-intron 5 (*G. gallus* chromosome 3, [36]), *26438* (*G. gallus* chromosome 3, [37]), βFib-intron5 (*G. gallus* chromosome 4, [38]), *15691* (*G. gallus* chromosome 5, [37]) and BRM-intron15 (*G. gallus* Z chromosome, [39]). PCR-amplifications were performed in a total volume of 10 µl with 10–20 ng of genomic DNA, GeneAmp 10× PCR Gold Buffer, 0.5 U of Taq polymerase (Roche), 2.0–2.5 mM MgCl_2_, 0.3 mM of each dNTP and primer concentrations of 0.15 µM. The thermocycling profile consisted of an initial denaturation step at 95°C for 3 min followed by 35 cycles at 95°C for 30 seconds, and a locus-specific annealing temperature of 55°C–60°C for 30 seconds, and 72°C for 30 seconds, with a final extension step at 72°C for 7 min. PCR products were cycle sequenced in both forward and reverse directions using the ABI BigDye Terminator Kit v3.1 (Applied Biosystems, Foster City, CA, USA) and then analyzed on an ABI 3730 automated sequencer. Sequences were edited and aligned using CodonCode Aligner v3.5.2 (CodonCode Corporation 2009) and Geneious Pro v5.4 (Biomatters Ltd 2011). Length polymorphisms were found in two nuclear loci: TGFb2 and *26438.* For TGFb2, we chose to remove them from the alignments prior to further analysis. For *26438*, because length polymorphism was located at the very end of the fragment we truncate the sequence data at the indel in the forward direction. All sequences have been submitted to GenBank (KF873022–KF873472).

#### Population structure and gene flow

Prior to analysis, the gametic phase was determined using PHASE 2.1 [40], [41]. The algorithm implemented in PHASE was run twice for each locus (10^4^ main iterations and 10^3^ burn-in, -x100 option) and the alleles with greatest probability (95% of the 386 individuals sequenced had posterior probability >90%) were used for subsequent analyses [42], [43]. The presence of recombination was tested by screening the sequences for possible breaking points using the GARD module [44] as implemented in HyPhy via the Web interface of DataMonkey [45]. The program DnaSP version 5.10.1 [46] was used to calculate a number of summary statistics: *θ* [47], haplotype diversity (Hd) [48], the number of segregating sites (S) [47] and one estimator of differentiation (K*st*; a haplotype based statistic similar to F*st*) [49]. Departures from a neutral model of molecular evolution across the entire brown scrub-robin sample and within each subspecies group was tested using the frequency spectrum of polymorphisms as measured by Tajima’s *D* (T*D*; [50]).

We used STRUCTURE v2.3 [51], [52] to identify groups of randomly mating individuals with different allele frequencies by minimizing deviations from Hardy-Weinberg expectations and linkage disequilibrium. The analysis was restricted to individuals that had missing data at no more than one locus (n = 63) using the *admixture* model with *correlated allele frequencies*. Because the algorithm implemented in STRUCTURE may have difficulty to detect hierarchical genetic subdivision we implemented the analysis within the subspecies *C. c. signata* to evaluate the extent of current subdivision. We ran five pseudo-replicates with 10^6^ Markov-Chain-Monte-Carlo iterations following a burn-in of 10^5^. Because the model implemented in STRUCTURE assumes no linkage disequilibrium, it was only run with the nuclear data. To test whether greater differentiation in the mtDNA was coincident with the groups recovered with nuclear data only, we used an analysis of molecular variance (AMOVA, [53]). The AMOVA was performed in ARLEQUIN v3.1 [54] and the statistical significance of the *F*-statistics was assessed using 10^5^ permutations.

Given the contemporary genetic structure and climatic niche differences (see results) we used the coalescent-based *isolation with migration* model, as implemented in the software IMa [55], [56] to test whether the boundary between the two subspecies has been impermeable since divergence, or whether gene flow has occurred. The IMa analysis was restricted to the populations closest to the area where the ranges of the subspecies presently abut and are though to have been persistent since the at least the Holocene: KZN (n = 12) and SLR (n = 10) populations. As a comparison, we also modelled migration between two populations within subspecies *C. c. signata.* These populations occupy two forests regions thought to have supported forest remnants during the LGM: KZN (n = 12) and PSJ (n = 16). Two separate analyses were performed: i) six nuclear loci and mtDNA locus and, ii) only six nuclear loci. After preliminary runs to determine the appropriate range of priors and confirm proper MCMC mixing, the final runs were performed with 30 coupled chains with a geometric heating scheme (g_1_ = 0.9 and g_2_ = 0.8) and allowed to continue for at least 3×10^6^ steps. The first 8×10^5^ steps were discarded as the burn-in. Parameter trend plots and ESS were used to assess convergence. Because the three types of marker are characterized by different effective population sizes, we included an inheritance scalar to adjust the parameters in the model: 0.25 for mitochondrial, 0.75 for Z-linked and 1.0 for autosomal loci. Time of divergence was obtained assuming a generation time of one year (as determined for its closest relative [57]) and a mean per locus mutation rate of 2.41×10^−8^ (derived from a neutral mutation rate of 1.35×10^−9^ substitutions/site/year for autosomal loci, 1.45×10^−9^ substitutions/site/year for the z-linked locus, and 1.35×10^−8^ substitutions/site/year for mtDNA; [58]).

## Results

### Ecological Axis of Morphological Divergence

**Figure 2:**
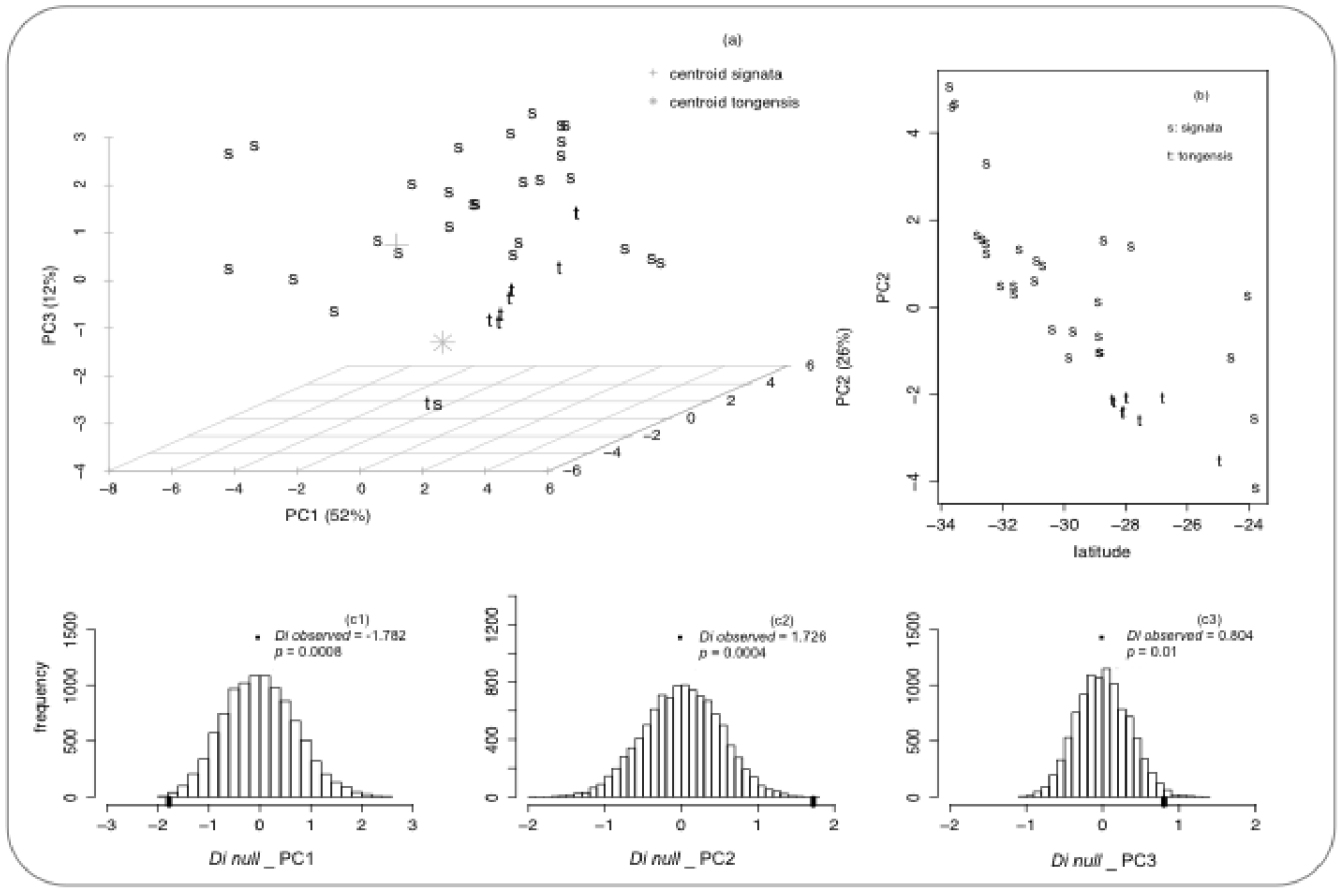
Climatic niche of the brown scrub-robin. (a) Principal component analysis on 19 climatic variables for the 36 occurrence points. (b) Multidimensional summary of climatic variation across the temperate-tropical gradient that runs along the south to north axis of the brown scrub-robin range, as quantified by PC2. (c1–3) Distribution of simulated values of *Di (Di_null)* under the null hypothesis of random occupation of the climatic niche measured for three principal components (PC1, PC2, PC3). The observed mean difference between *C. s. signata* and *C. s*. *tongensis (Di observed*) is plotted as a black mark on the null distribution.

The first three principal components accounted for 91% of the variance in the climatic data that characterize the range of the brown scrub-robin: PC1, PC2 and PC3 explained 53%, 26% and 12% (Figure 2A), respectively. The first axis (PC1) correlated positively with minimum temperature of the coldest month (0.301), precipitation of the coldest quarter (0.295) and precipitation of the driest quarter (0.292); PC2 associated positively with mean temperature of the wettest quarter (0.365) and maximum temperature of the warmest month (0.327) and the third axis (PC3) was associated with annual precipitation (−0.482). PC1 reflects a major difference in precipitation between the cooler highlands of Limpopo and KwaZulu-Natal and the warmer eastern coastal lowlands, regardless of latitude. PC2 describes a tropical to temperate climatic gradient with PC2 scores decreasing towards the southern portion of the species range (highest latitude; Figure 2B).

**Figure 3:**
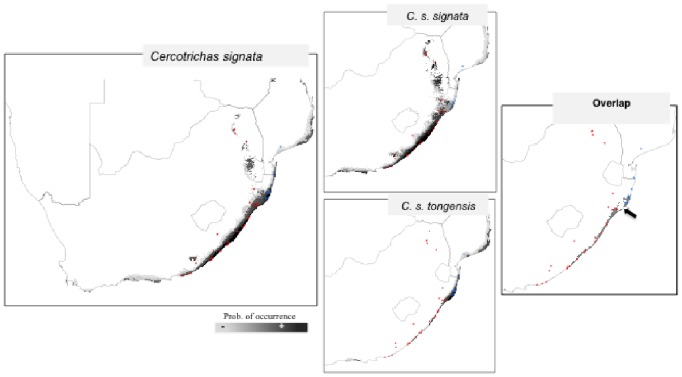
Geographical projection of the climatic niche model of *Cercotrichas signata* and the two subspecies: *C. s. signata* and *C. s. tongensis* including a region of predicted overlap. The arrow in panel ‘overlap’ emphasizes a geographical region of niche dissimilarity and coincides with the subspecies boundary (see Figure 1).

The geographical projection of the climatic niches of each subspecies revealed areas of reciprocal suitability, i.e. niche overlap. Some portion of the geographical range of *signata* was predicted as climatically suitable for *tongensis* and vice-versa (Figure 3). The models also predicted a small area (rough linear distance of 50 km), south of the St. Lucia region, where the occurrence of either subspecies is unlikely (Figure 3– arrow in panel ‘overlap’). In parallel, our multivariate approach revealed that the two subspecies occupy significantly different portions of the niche than would be expected under the null distribution. The values of *Di observed* for PC1 = −1.782, PC2 = 1.726 and PC3 = 0.801 were significantly larger than the *Di null* (p<0.01; Figure 2 C1–C3). A MANOVA analysis, using the first three PCA axes as dependent variables and subspecies as categorical variables, revealed significant differences in the climatic envelopes of the two subspecies (Wilks = 0.314, *p*<0.001). When comparing the niche differentiation within *signata* only the *Di observed* for PC1 was significantly larger than the *Di null* (*p*<0.01).

We found sexual dimorphism for all morphological variables quantified (mass: *F = *8.025, *p = *0.006; wing: *F* = 49.072, *p*<0.001; tarsus: *F* = 17.690, *p*<0.001; size: *F* = 16.3728, *p*<0.001). Furthermore, the ANOVA revealed significant differences in all traits between birds from different geographical areas (Table S3). The interaction sex*geography was non-significant for all traits quantified, indicating that females were smaller than males regardless of geographical location. Explicitly testing for differences between subspecies, we found that *signata* and *tongensis* differ in mass (*F* = 124.167, *p*<0.001), wing-length (*F* = 34.707, *p*<0.001) and structural size (*F* = 35.889, *p*<0.001), but not in tarsus-length (*F* = 1.422, *p* = 0.236).

All morphological variables except tarsus-length were significantly and positively correlated with climate in a linear relationship (Table 1). Yet, the correlation with PC1 (precipitation seasonality) was relatively weak and explained little of the variation in the data. Wing-length, mass and size were strongly correlated with the temperate-tropical climate gradient quantified by PC2. Comparing mean AICc among models revealed that the model including a quadratic factor testing the effect of PC2 explained more of the variability in mass, wing and structural body size than the other three models. Delta AICc for alternative models were greater than 14.0. For tarsus, the model testing the effect of PC1 with a quadratic factor is better supported (AICc weight = 0.59) although Delta AICc for alternative models was smaller or equal to 3.09 (Table 1). This indicates that, rather than changing gradually along the gradient, morphology is affected to a greater extent by extreme conditions and thus best explained by a quadratic function. Birds in the tropical localities of Maputaland (*tongensis*) were smaller than birds from the subtropical and temperate portion of the range (*signata;* see Figure S1).

**Table 1:** The effect of climate on morphological traits.

Trait	Model	R^2^	*p*	K	Delta_AICc	AICc weight
**Mass**						
	∼PC1	0.037	0.08	3	63.43	0
	∼PC1+ PC1^2^	0.149	<0.01	4	55.61	0
	∼PC2	0.202	<0.01	3	48.20	0
	∼PC2+ PC2^2^	0.572	<0.001	4	0	1
**Tarsus**						
	∼PC1	0.008	0.413	3	2.70	0.15
	∼PC1+ PC1^2^	0.066	0.067	4	0	0.59
	∼PC2	0.037	0.587	3	3.09	0.13
	∼PC2+ PC2^2^	0.032	<0.01	4	2.99	0.13
**Wing**						
	∼PC1	0.065	0.02	3	19.86	0
	∼PC1+ PC1^2^	0.131	<0.001	4	16.10	0
	∼PC2	0.091	<0.01	3	17.55	0
	∼PC2+ PC2^2^	0.288	<0.001	4	0	1
**Size**						
	∼PC1	0.058	0.03	3	18.38	0
	∼PC1+ PC1^2^	0.083	0.03	4	18.36	0
	∼PC2	0.097	<0.01	3	14.91	0
	∼PC2+ PC2^2^	0.269	<0.001	4	0	1

PC1 illustrates precipitation differences between cooler and warmer sites, PC2 explains the tropical-temperate climatic gradient. The non-linear relationship between morphology and climate was modelled by including a quadratic term.

K: number of parameters in the model.

### Ecological Axis of Genetic Divergence

Estimates of polymorphism (S, Hd, *θ*) and divergence (K*st*) for mtDNA and nDNA sequence data are summarized in Table 2. Levels of nucleotide polymorphism among loci were heterogeneous: values of *S* varied from 0.001 to 0.01. Genetic differentiation at the mtDNA genes among localities, K*st*, was high (average 62%; 999 permutations: *P*<0.001). For the six nuclear introns, significant differentiation was also recovered: K*st* values varied from 21% to 69%. Overall, we found deviations from neutral expectations of evolution for mtDNA (T*D* = 3.018; Table 2). This positive value indicates that despite little allelic diversity, the variable alleles exist at high frequencies; a pattern expected under spatially divergent selection. When restricting the analysis to each subspecies, T*D* was only significantly different from neutral expectations for two autosomal loci in *signata* (Table 2).

**Table 2:** Population genetic descriptive statistics by subspecies. n: number of alleles; bp: base pairs; S: segregating sites; Hd: haplotype diversity; θ^#^: theta per site; Kst: genetic divergence (*p<0.01, **p<0.001).

				*signata*				*tongensis*				Overall	
Genome		Locus (n)	Sites (bp)	S	Hd	θ^#^ (SE)	T*D*	S	Hd	θ^#^ (SE)	T*D*	T*D*	Kst
mtDNA		ATPase6-8 (74)	800	11	0.846	0.0311 (0.001)	−1.267	7	0.709	0.0028 (0.001)	0.194	3.018**	0.643**
nDNA	Autosomal	GAPDH (128)	327	2	0.453	0.001 (0.001)	1.272	6	0.480	0.004 (0.002)	−1.114	0.1289	0.577**
		FIB5 (126)	516	4	0.506	0.002 (0.001)	0.464	8	0.603	0.004 (0.01)	−1.124	1.123	0.695**
		15691 (122)	580	2	0.519	0.001 (0.001)	−1.311*	6	0.074	0.002 (0.001)	−0.734	−0.661	0.679**
		TGFb2 (128)	566	4	0.517	0.014 (0.001)	−0.564	6	0.628	0.024 (0.001)	−0.828	−1.331	0.211**
		26438 (120)	419	9	0.306	0.0044 (0.002)	−2.088*	9	0.455	0.005 (0.002)	−0.468	−0.913	0.628**
	Z -linked	BRM (148)	299	4	0.197	0.003 (0.001)	−1.420	2	0.542	0.002 (0.001)	0.603	−0.693	0.618**

**Figure 4:**
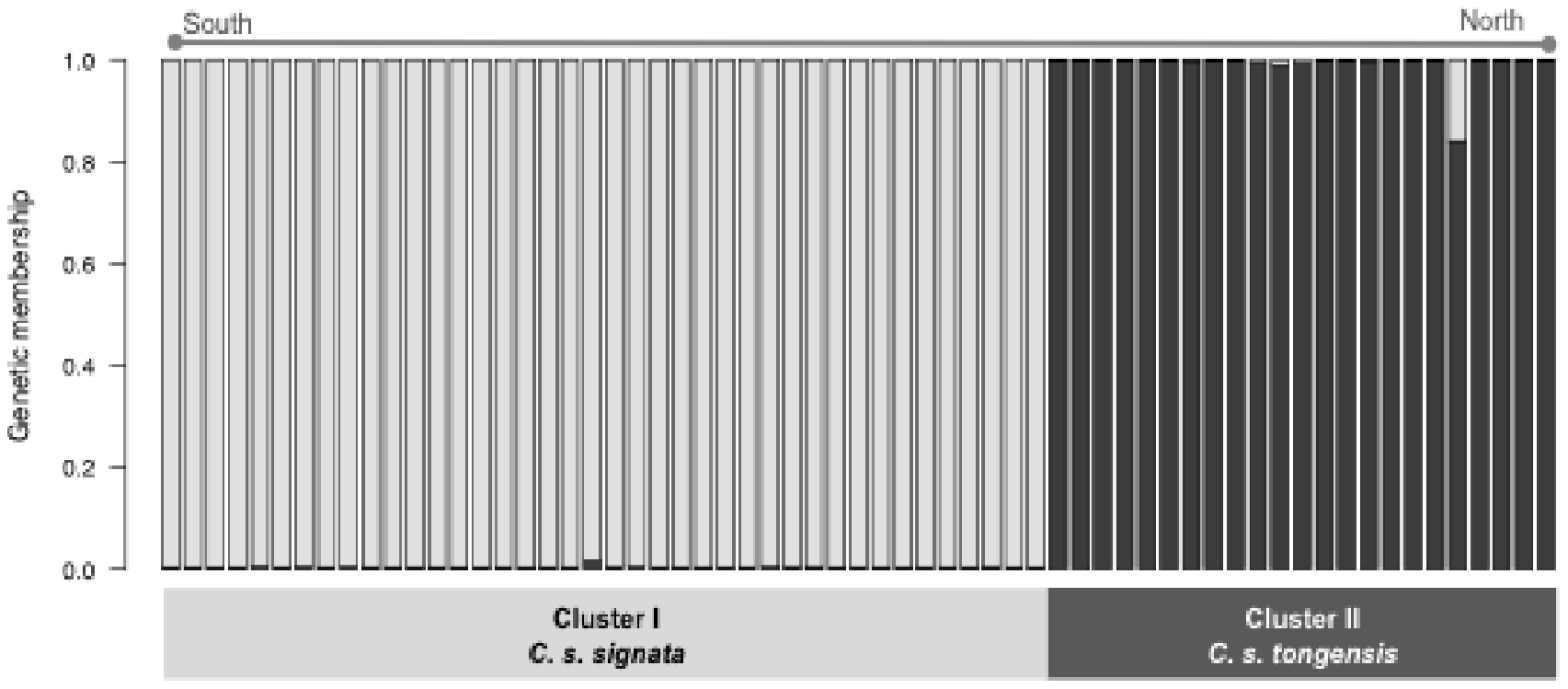
Results from Bayesian clustering analysis of genotypic data (six nuclear introns) showing two distinct gene pools (LnP (K = 2) = −663.5) corresponding to the two subspecies: *C. s. signata* (cluster I) and *C. s. tongensis* (cluster II).

The SPB and GARD algorithms did not detect any evidence of recombination events. Thus, all the subsequent analyses were performed using full sequences. The Bayesian clustering analysis of nuclear genotypes revealed two distinct nuclear clusters (LnP (K = 1) =  −1043.84±30.78 *vs.* LnP (K = 2) =  −663.5±53.72) with strong geographical affinities. Members of *cluster I* were all from subtropical and temperate areas (*signata*) and members of *cluster II* were all from tropical Maputaland (*tongensis*) with no evidence of recent introgression having occurred (Figure 4). All but one individual were assigned to one of the clusters with a probability of membership higher than 0.980 across the five STRUCTURE runs. The exception, one bird sampled in the Maputaland region, had a mean probability of membership of 0.842±0.023 across five runs. Implementing the analysis within *C. c. signata* revealed no clear signal of subdivision (LnP (K = 1) =  −342.12, Var (LnP) = 13.64 *vs*. LnP (K = 2) =  −341.86, Var (LnP) = 55.38). Genetic differentiation along the costal transect peaked between populations in KZN and those south of SLR (K*st* nuclear = 0.539), and between PSJ and KZN (K*st* nuclear = 0.292).

Using STRUCTURE membership to define two major genetic groups, the AMOVA implemented with the mitochondrial data revealed the same strong geographical structure: the majority of the mitochondrial genetic variation occurred between subspecies (96%; F_CT = _0.962, *p*<0.001) with only a smaller portion attributed to among populations within subspecies (3.27%; F_SC_ = 0.117, *p* = 0.008). Because of this strikingly high differentiation between groups living in tropical versus sub-tropical-temperate forests (40 fixed differences; 4.3% sequence divergence) and the fact that the mtDNA loci examined are protein coding, we also analysed the amino acid profiles. This revealed that of the seven non-synonymous mutations found at ATP synthase subunit 6, five amino acids segregate between the two nuclear genetic clusters and coincide with the currently defined subspecies boundaries (Figure S2).

The IMa results revealed different trends in the amount of gene flow detected between the two groups of populations we compared (SLR*_tongensis_* vs. KZN*_signata_*; KZN*_signata_* vs. PSJ*_signata_*), as well as that the estimates of gene flow vary in response to whether or not the mtDNA data was included in the analyses (Table 3). Migration rates between contiguous populations of the two subspecies were estimated to be zero: *m_KZN − SLR = _*0.0015 (90% High Posterior Density: 0.0015–1.4535) and *m_SLR – KZN = _*0.0015 (90% HPD: 0.0015–0.4275). Note that 0.0015 was the first bin of the prior and therefore corresponds to zero in both directions. When removing the mtDNA from the data set, the estimates of migration were slightly greater (Table 3). Introgression from KZN*_signata_* into SLR*_tongensis_* was higher than in the opposite direction. However, the 90% high posterior densities included zero (0.001 was the first bin). In contrast, migration between populations within subspecies *signata* was nearly 13 times larger than between subspecies populations (Table 3). Excluding mtDNA from the data set had a major effect on the directionality of gene flow. Migration southward, from KZN to PSJ, became significantly different from zero (*m_KZN – PSJ = _*2.783; 90% HPD: 0.203–13.403) indicating that nuclear gene flow between these two populations occurs in both directions (Table 3).

**Table 3:** Gene flow estimates: (1) between subspecies: populations in the coastal forests of the St. Lucia Region (SLR) and KwaZulu-Natal scarp forest (KZN); and (2) within subspecies *C. s. signata*: populations in the KZN scarp forests (KZN) and Port St. Johns coastal scarp forests (PSJ).

Populations	Analysis	*m* (HiPt)	90% HPD
(1)	Seven loci	*m*_SLR−KZN = _0.0015	0.0015–0.4275
SLR vs. KZN	(mtDNA+nuclearDNA)	*m*_KZN−SLR = _0.0015	0.0015–1.4535
	Six loci	*m*_SLR−KZN = _0.0010	0.0010–0.1690
	(nuclear DNA)	*m*_KZN−SLR = _0.3210	0.0010–0.9730
(2)	Seven loci	*m*_KZN−PSJ = _0.0075	0.0075–12.5475
KZN vs. PSJ	(mtDNA+nuclearDNA)	*m*_PSJ−KZN_ = 13.525	5.4750–30.5250
	Six loci	*m*_KZN−PSJ = _2.7825	0.2025–13.4025
	(nuclear DNA)	*m*_PSJ−KZN_ = 20.4750	9.2750–41.4750

HiPt: highest point of the posterior density distribution. HPD: interval containing 90% of the posterior density interval.

## Discussion

**Figure 5:**
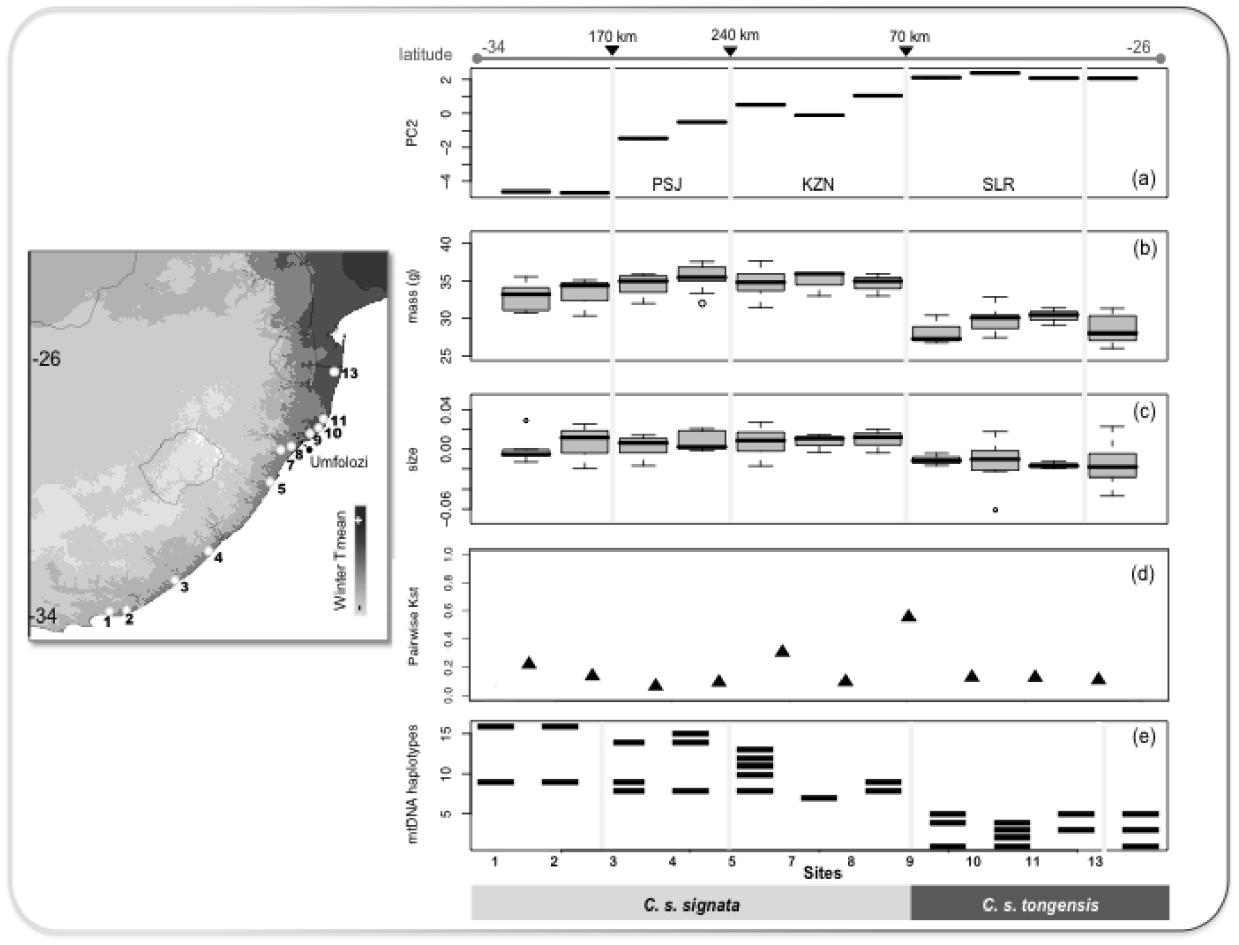
Variation in climate (a), mass (b), size (residuals of wing-length on tarsus length) (c), pairwise nuclear K*st* (d) and, mtDNA haplotypes (e) along the temperate to tropical transect (latitude −36 to −24) sampled across the range of the brown scrub-robin. K*st* is a pairwise measure of divergence; therefore, it is represented as the midpoint between two adjacent sites. Geographical distances, in km, between the main forest blocks referred to in the text are annotated at the top. The side map depicts the geographical variation in mean temperature during winter (WinterTmean = Bio11: mean temperature of the coldest quarter; light grey represents lower temperatures and dark grey higher temperatures).

The conceptual perspective of speciation as a gradual accumulation of genetic differences and reproductive isolation provides a useful framework for investigating the factors contributing to partial reproductive isolation between ecologically divergent populations. By considering the eco-geographical, morphological and genetic context of current populations of the brown scrub-robin we revealed populations positioned at different stages along the speciation continuum: i) continuous variation without reproductive isolation in populations of *signata* that currently inhabit the temperate and sub-tropical forests that were historically spatially isolated, and ii) strong multidimensional differentiation with marked reproductive isolation between *signata* and *tongensis* across divergent environments (summarized in Figure 5).

Exactly how divergence was attained remains unclear. Divergence between *tongensis* and *signata* might have been initiated in different environments with natural selection promoting adaptive divergence and thereby impeding gene flow; or it might have started via the reduction of migration and consequently gene flow between populations in similar environments, after which natural selection increased the frequency of phenotypes with greater fitness. In contrast, the lack of morphological and genetic divergence, and the evidence of extensive gene flow estimated between populations of *signata* currently occupying central and southern forests that were historically isolated (KZN and PSJ, Figure 1), and which occur in distinct portions of the climatic gradient (Figure 5a–e), revealed that changes in selective pressures at the end of the Last Glacial Maximum promoted the reestablishment of migration and hence the reversal of divergence between the two southern temperate populations (KZN and PSJ).

### Ecological Determinants of the Divergence Process

Since the early 20^th^ century, climate has been regarded as the primary factor that affects the density and geographical distribution of populations [59], [60], and thereby ultimately promotes speciation (e.g. [61] mechanism of thermal adaptation). Thus, quantifying how climatic niches of populations change across space and time is a fundamental step in speciation research. We predicted niche similarity to facilitate gene flow, whereas niche divergence would impede gene exchange. Our results of the test that compared the amount of climatic divergence to the null expectation of random occupation of the climatic niche (Figure. 2C) and estimates of migration (Table 3) corroborated those expectations. Interestingly, the geographical projections of the climatic niche models revealed that a portion of the range of *signata* is suitable for *tongensis*, and vice-versa. However, the overlap is disrupted in the region that coincides with the current range boundary between *signata* and *tongensis.* This indicates that the current geographical isolation is a consequence of climatic differences and thereby eco-geographically isolated. The finding that dissimilarity in climatic niches, as quantified in PC2 (representing a temperature gradient) but not PC1 (gradient of winter mean temperature and precipitation), is associated with a lack of gene flow as well as with morphological divergence further strengthens the previous argument and highlights the role of niche divergence as a diversifying force that has promoted reproductive isolation in this system.

The significant difference in structural body size and body mass found along the climatic axis representing the temperate to tropical gradient, with smaller birds occupying warmer climates, suggests these traits have diverged as a consequence of physiological adaptation to varying climates across the species range. Variation in body size can be explained by selection on thermoregulatory capabilities: larger-bodied individuals loose heat more slowly, and thus have greater fitness in cooler climates [62] whereas smaller body size reduces heat loading, which is advantageous in warmer climates [63]. Beside local adaptation, phenotypic divergence can be due to phenotypic plasticity, i.e. be under direct influence of environmental factors; but this seems unlikely to be the sole explanation in our system because the traits in which the two taxa diverge tend to have high heritability [64].

Morphological divergence can also be explained by factors other than abiotic variables. Divergence can occur between populations, if traits involved in social interactions increase the mating success of some individuals [65], [66]. The two subspecies of brown scrub-robin differ in plumage colour and white patterning on the throat, with *signata* being larger and darker with narrower white markings than *tongensis*. Therefore, it is possible that a trait (e.g., size, plumage colouration) involved in social interactions, such as mate attraction or competition with same-sex conspecifics, increased in frequency as gene flow progressively diminished. This sub-component of natural selection can promote assortative mating which, in turn, may lead to irreversible reproductive isolation. Song is another trait that could have facilitated the process of divergence [67], [68], and may co-vary with morphology. Although the difference between the song structures of the two forms is considered subtle [69], the influence of song on the divergence process remains to be tested.

### Speciation in the Forests of Southeast Africa

Poynton (1961; [70]) first described a gradual north-to-south impoverishment of tropical flora and fauna along the southeastern coast of Africa (the ‘subtropical subtraction effect’), with a major loss of tropical elements occurring around the St. Lucia Estuary and Umfolozi Swamp (Figure 1). This area is considered a biogeographic transition zone [71], [72]. Geological data indicate this estuarine barrier developed in the late Holocene (4,000 ybp; Scoot 1982 in [17]). While climatic oscillations during the Quaternary may have been the major cause for this abrupt loss of biodiversity [17], [18] it may also be related to the position of the 18°C surface isotherm in the eastern coast of southern Africa [69], [72].

Our estimate of the time of divergence between *tongensis* and *signata* is centred on the late Pliocene (3 million years ago; 90% HPD: 403,427–25,550,390 ybp), which suggests that divergence in the brown scrub-robin was not triggered by the formation of an estuarine barrier. Populations currently inhabiting the scarp forests of KwaZulu-Natal (*signata*) and the coastal Indian Ocean forest of the St. Lucia region (*tongensis*) have been following distinct evolutionary trajectories, with no current exchange of migrants between them. In contrast, there is a lack of current population genetic structure and extensive gene flow between populations of *signata* occupying the coastal scarp forest of the Eastern Cape (PSJ) and the southern coastal and northern interior scarp forests of KwaZulu-Natal (KZN). Furthermore, gene flow predominantly occurs from south to north (*m_KZN – PSJ = _*2.782 vs *m_PSJ – KZN_ = *20.475) suggesting that the brown scrub-robin populations from the temperate forests might have a broader climatic niche and thus be more tolerant of subtropical conditions.

Although it is difficult to demonstrate a causative effect of the present morphological differences observed between the two subspecies as the driver of the divergence process, morphology likely co-varies with causal traits that maintain reproductive isolation. However, only by concentrating future work in the archipelago of small forest patches at the boundary between the two subspecies will we be able to fully understand whether reproductive isolation is a consequence of divergent selection on physiological tolerances and/or sexual selection on plumage, size or song.

Gaining a population-level perspective is essential if we are to understand how ecological and micro-evolutionary processes interact to affect the continuous process of species formation. In turn, this basic knowledge about the origin of species is relevant to our understanding of why some areas, such as the understudied southern African Maputaland-Pondoland-Albany region, harbour such high species richness.

## Supporting Information

Figure S1 **Dimorphism in quantitative traits measured across in the brown scrub-robin range. Triangles represent** ***C. s. tongensis*** **and circles C. s.** ***signata*** **. Sex is colour coded.**
(TIFF)

Figure S2 **Amino acid changes in the ATP synthase subunit 6, which is encoded in the mtDNA genome.** Each individual vertical line represents one individual and the colour code (black vs red) one of the two amino acids observed. E.g., at position 14 all individuals of subspecies *tongensis* have a Leucine whereas all individuals from *signata* have a phenylalanine. Five of the seven amino acid changes segregate in concert with morphology, climate and nuclear genotypes. L: Leucine, F: Phenylalanine, M: Methionine, T: Threonine, N: Aspargine, S: Serine, A: Alanine, V: Valine, I: Isoleucine.
(TIFF)

Table S1 **Point locality data with details whether morphological and genetic data was collected and the respective samples sizes.** Numbering code used in Figure 1 and Figure 5. Latitude and longitude reported in decimal degrees.
(DOC)

Table S2 **Morphological data: tarsus-length (mm), wing-length (mm; from the carpal joint to the tip of the longest primary feather) and body mass (g).** The sex of all birds was determined using a PCR-based assay. Please, see Methods section for further details.
(DOC)

Table S3 **Effect of geography and sex on morphological variation in the brown scrub-robin as revealed by a two-way ANOVA. N_females_ = 29, N_males_ = 52.**
(DOC)

File S1 **Multivariate approach to test niche overlap – R script.**
(DOC)
