# Supplementary figures and images for: The Ecological and Geographic Context of Morphological and Genetic Divergence in an Understorey-Dwelling Bird
Source: PLoS One. 2014 Feb 7;9(2):e85903. doi: 10.1371/journal.pone.0085903 (PMC3917827; doi:10.1371/journal.pone.0085903)

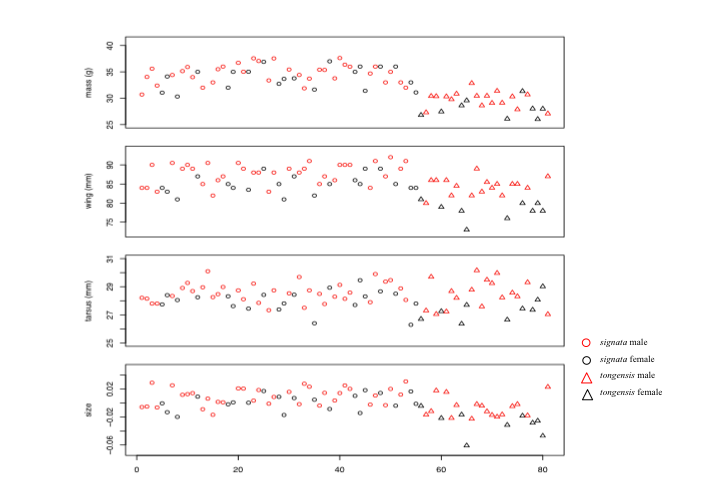

Supplement: Figure S1 — Dimorphism in quantitative traits measured across in the brown scrub-robin range. Triangles represent C. s. tongensis and circles C. s. signata . Sex is colour coded. (TIFF) [file pone.0085903.s001.tif]

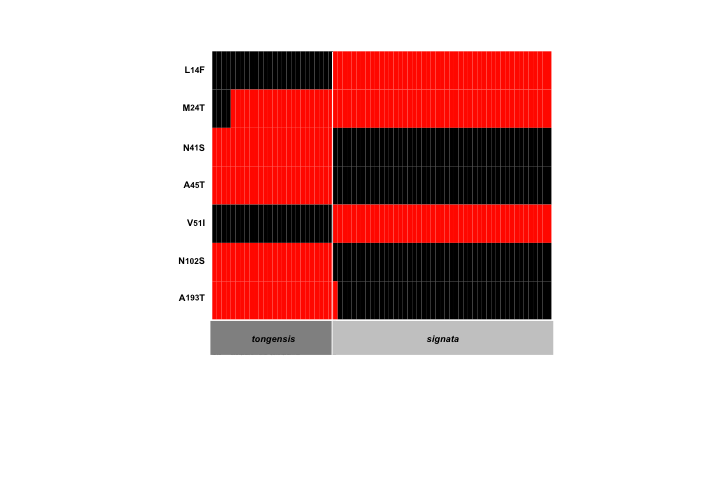

Supplement: Figure S2 — Amino acid changes in the ATP synthase subunit 6, which is encoded in the mtDNA genome. Each individual vertical line represents one individual and the colour code (black vs red) one of the two amino acids observed. E.g., at position 14 all individuals of subspecies tongensis have a Leucine whereas all individuals from signata have a phenylalanine. Five of the seven amino acid changes segregate in concert with morphology, climate and nuclear genotypes. L: Leucine, F: Phenylalanine, M: Methionine, T: Threonine, N: Aspargine, S: Serine, A: Alanine, V: Valine, I: Isoleucine. (TIFF) [file pone.0085903.s002.tif]
