# Supplementary material for: The Ecological and Geographic Context of Morphological and Genetic Divergence in an Understorey-Dwelling Bird
Source: PLoS One. 2014 Feb 7;9(2):e85903. doi: 10.1371/journal.pone.0085903 (PMC3917827; doi:10.1371/journal.pone.0085903)
Supplement: Table S1 — Point locality data with details whether morphological and genetic data was collected and the respective samples sizes. Numbering code used in Figure 1 and Figure 5. Latitude and longitude reported in decimal degrees. (DOC) [file pone.0085903.s003.doc]

| **Longitude** | **Latitude** | **Subspecies** | **Locality Name** | **Morphology** | **Genetic** | **Figure 1 and 5** |
| --- | --- | --- | --- | --- | --- | --- |
| 26.77886 | -33.64837 | *signata* | Kasouga | 6 | 5 | 1 |
| 27.01436 | -33.54209 | *signata* | Kleinmonde | 3 | 4 | 2 |
| 28.40416 | -32.66250 | *signata* | Fish River Mouth |  | 1 | 3 |
| 28.45215 | -32.53950 | *signata* | Morgan’s Bay | 7 | 7 | 3 |
| 29.06420 | -32.06750 | *signata* | Mboyte | 10 | 10 | 4 |
| 30.67588 | -30.38909 | *signata* | Pennington | 20 | 9 | 5 |
| 31.68988 | -28.83523 | *signata* | Ongoye |  | 2 | 8 |
| 31.42168 | -27.82411 | *signata* | Ngome | 2 | 2 | 12 |
| 31.13045 | -28.72311 | *signata* | Nkandhla | 1 | 1 | 6 |
| 31.37773 | -28.88671 | *signata* | Entumeni | 2 | 2 | 7 |
| 31.64924 | -28.85407 | *signata* | Ongoye | 3 | 3 | 8 |
| 30.01633 | -23.81247 | *signata* | Magoebaskloof | 1 | 1 | 14 |
| 26.41670 | -33.71670 | *signata* | Alexandria Forest |  |  |  |
| 28.40218 | -32.66244 | *signata* | Kei Mouth |  |  |  |
| 31.45000 | -28.86670 | *signata* | Eshowe |  |  |  |
| 29.53330 | -31.63330 | *signata* | Port St. Johns |  |  |  |
| 31.06670 | -29.71670 | *signata* | Umhlanga Rocks |  |  |  |
| 30.28330 | -30.70000 | *signata* | Oribi Gorge Nature Reserve |  |  |  |
| 30.06670 | -23.78330 | *signata* | Woodbush Forest Reserve |  |  |  |
| 30.88330 | -24.58330 | *signata* | Mariepskop Forest Reserve |  |  |  |
| 27.21670 | -32.83330 | *signata* | Pirie |  |  |  |
| 29.55000 | -31.61670 | *signata* | Mutafufu River |  |  |  |
| 29.63330 | -31.45000 | *signata* | Lusikisiki |  |  |  |
| 31.01670 | -29.85000 | *signata* | Durban |  |  |  |
| 30.08330 | -24.03330 | *signata* | Wolkberg Forest |  |  |  |
| 27.35000 | -32.51670 | *signata* | Kologha Forest |  |  |  |
| 28.58330 | -32.51670 | *signata* | Gqunqe River |  |  |  |
| 28.88330 | -30.90000 | *signata* | Amanzamnyama Forest |  |  |  |
| 30.23330 | -30.96670 | *signata* | Munster |  |  |  |
| 32.84721 | -26.78239 | *tongensis* | Kosi Bay | 10 | 10 | 13 |
| 32.43206 | -27.96196 | *tongensis* | Santa Lucia | 3 | 3 | 11 |
| 32.43843 | -28.06458 | *tongensis* | Cape Vidal | 10 | 8 | 10 |
| 32.26838 | -28.43492 | *tongensis* | Futululu | 3 | 6 | 9 |
| 33.28330 | -24.95000 | *tongensis* | Chimonzo |  |  |  |
| 32.45000 | -28.10000 | *tongensis* | Fanies Island |  |  |  |
| 32.66670 | -27.55000 | *tongensis* | Sodwama Bay |  |  |  |
| 32.41670 | -28.38330 | *tongensis* | Santa Lucia |  |  |  |
