# Supplementary material for: The Ecological and Geographic Context of Morphological and Genetic Divergence in an Understorey-Dwelling Bird
Source: PLoS One. 2014 Feb 7;9(2):e85903. doi: 10.1371/journal.pone.0085903 (PMC3917827; doi:10.1371/journal.pone.0085903)
Supplement: Table S2 — Morphological data: tarsus-length (mm), wing-length (mm; from the carpal joint to the tip of the longest primary feather) and body mass (g). The sex of all birds was determined using a PCR-based assay. Please, see Methods section for further details. (DOC) [file pone.0085903.s004.doc]

| **Extraction** | **Locality** | **Tarsus (mm)** | **Wing (mm)** | **Mass (g)** | **Sex** | **Subspecies** |
| --- | --- | --- | --- | --- | --- | --- |
| **CsiKSG01** | Kasouga | 28.21 | 84.0 | 30.7 | M | *signata* |
| **CsiKSG02** | Kasouga | 28.15 | 84.0 | 34.04 | M | *signata* |
| **CsiKSG03** | Kasouga | 27.80 | 90.0 | 35.6 | M | *signata* |
| **CsiKSG04** | Kasouga | 27.81 | 83.0 | 32.4 | M | *signata* |
| **CsiKSG05** | Kasouga | 27.74 | 84.0 | 31.05 | F | *signata* |
| **CsiKSG06** | Kasouga | 28.40 | 83.0 | 34.12 | F | *signata* |
| **CsiKNM01** | Kleinemonde | 28.34 | 90.5 | 34.4 | M | *signata* |
| **CsiKNM03** | Kleinemonde | 28.05 | 81.0 | 30.32 | F | *signata* |
| **CsiKNM04** | Kleinemonde | 28.91 | 89.0 | 35.14 | M | *signata* |
| **CsiMRG01** | Morgan’s Bay | 29.27 | 90.0 | 35.92 | M | *signata* |
| **CsiMRG02** | Morgan’s Bay | 28.69 | 89.0 | 34.0 | M | *signata* |
| **CsiMRG03** | Morgan’s Bay | 28.25 | 87.0 | 35.0 | F | *signata* |
| **CsiMRG04** | Morgan’s Bay | 28.96 | 85.0 | 32.0 | M | *signata* |
| **CsiMRG05** | Morgan’s Bay | 30.10 | 90.5 | 43.5 | M | *signata* |
| **CsiMRG06** | Morgan’s Bay | 28.26 | 82.0 | 33.0 | M | *signata* |
| **CsiMRG07** | Morgan’s Bay | 28.47 | 86.0 | 35.5 | M | *signata* |
| **CsiMBT01** | Mbotye | 28.98 | 87.0 | 36.0 | M | *signata* |
| **CsiMBT02** | Mbotye | 28.32 | 85.0 | 32.0 | F | *signata* |
| **CsiMBT03** | Mbotye | 27.62 | 84.0 | 35.0 | F | *signata* |
| **CsiMBT04** | Mbotye | 28.74 | 90.5 | 36.71 | M | *signata* |
| **CsiMBT06** | Mbotye | 28.11 | 89.0 | 35.02 | M | *signata* |
| **CsiMBT07** | Mbotye | 27.44 | 83.5 | 35.02 | F | *signata* |
| **CsiMBT08** | Mbotye | 29.22 | 88.0 | 37.57 | M | *signata* |
| **CsiMBT09** | Mbotye | 27.85 | 88.0 | 37.06 | M | *signata* |
| **CsiMBT10** | Mbotye | 28.42 | 89.0 | 36.9 | F | *signata* |
| **CsiMBT11** | Mbotye | 27.33 | 83.0 | 33.38 | M | *signata* |
| **CsiPNT01** | Pennington | 29.69 | 88.0 | 34.41 | M | *signata* |
| **CsiPNT02** | Pennington | 27.50 | 89.0 | 31.88 | M | *signata* |
| **CsiPNT03** | Pennington | 28.74 | 88.0 | 37.55 | M | *signata* |
| **CsiPNT04** | Pennington | 27.38 | 85.0 | 32.73 | F | *signata* |
| **CsiPNT05** | Pennington | 27.81 | 81.0 | 33.67 | F | *signata* |
| **CsiPNT06** | Pennington | 28.53 | 89.0 | 35.44 | M | *signata* |
| **CsiPNT07** | Pennington | 28.43 | 87.0 | 33.77 | F | *signata* |
| **CsiPNT08** | Pennington | 28.74 | 91.0 | 33.72 | M | *signata* |
| **CsiPNT09** | Pennington | 26.40 | 82.0 | 31.64 | F | *signata* |
| **CsiPNT10** | Pennington | 28.49 | 85.0 | 35.41 | M | *signata* |
| **CsiPNT11** | Pennington | 27.77 | 87.0 | 35.36 | M | *signata* |
| **CsiPNT12** | Pennington | 28.93 | 85.0 | 37.0 | F | *signata* |
| **CsiPNT13** | Pennington | 28.29 | 86.0 | 33.77 | M | *signata* |
| **CsiPNT14** | Pennington | 29.13 | 90.0 | 37.62 | M | *signata* |
| **CsiPNT15** | Pennington | 28.14 | 90.0 | 36.36 | M | *signata* |
| **CsiPNT16** | Pennington | 28.58 | 90.0 | 35.98 | M | *signata* |
| **CsiPNT17** | Pennington | 27.70 | 86.0 | 35.0 | F | *signata* |
| **CsiPNT18** | Pennington | 29.46 | 85.0 | 36.0 | F | *signata* |
| **CsiPNT19** | Pennington | 28.31 | 89.0 | 31.4 | F | *signata* |
| **CsiPNT20** | Pennington | 27.90 | 84.0 | 34.7 | M | *signata* |
| **CsiNKD01** | Nkandla | 29.90 | 91.0 | 36.0 | M | *signata* |
| **CsiETM01** | Entumeni | 28.67 | 89.0 | 36.0 | F | *signata* |
| **CsiETM02** | Entumeni | 29.36 | 87.0 | 33.0 | M | *signata* |
| **CsiNGY03** | Ongoye | 29.47 | 92.0 | 35.0 | M | *signata* |
| **CsiNGY04** | Ongoye | 28.50 | 85.0 | 36.0 | F | *signata* |
| **CsiNGY05** | Ongoye | 28.88 | 89.0 | 33.0 | M | *signata* |
| **CsiNGM01** | Ngome | 28.06 | 91.0 | 32.0 | M | *signata* |
| **CsiNGM02** | Ngome | 26.29 | 84.0 | 33.0 | F | *signata* |
| **CsiMGK01** | Magoebaskloof | 27.80 | 84.0 | 31.1 | F | *signata* |
| **CsiFTL02** | Futululu | 26.70 | 81.0 | 26.8 | F | *tongensis* |
| **CsiFTL03** | Futululu | 27.30 | 80.0 | 27.3 | M | *tongensis* |
| **CsiFTL04** | Futululu | 29.70 | 86.0 | 30.4 | M | *tongensis* |
| **CsiCPV01** | Cape Vidal | 27.05 | 86.0 | 30.36 | M | *tongensis* |
| **CsiCPV02** | Cape Vidal | 27.25 | 79.0 | 27.46 | F | *tongensis* |
| **CsiCPV03** | Cape Vidal | 27.23 | 86.0 | 30.32 | M | *tongensis* |
| **CsiCPV04** | Cape Vidal | 28.68 | 82.0 | 29.79 | M | *tongensis* |
| **CsiCPV05** | Cape Vidal | 28.20 | 84.5 | 30.84 | M | *tongensis* |
| **CsiCPV06** | Cape Vidal | 26.36 | 78.0 | 28.62 | F | *tongensis* |
| **CsiCPV07** | Cape Vidal | 27.7 | 73.0 | 29.57 | F | *tongensis* |
| **CsiCPV08** | Cape Vidal | 28.78 | 82.0 | 32.85 | M | *tongensis* |
| **CsiCPV09** | Cape Vidal | 30.16 | 89.0 | 30.44 | M | *tongensis* |
| **CsiCPV10** | Cape Vidal | 27.59 | 83.0 | 28.62 | M | *tongensis* |
| **CsiSTL01** | Santa Lucia | 29.49 | 85.5 | 30.45 | M | *tongensis* |
| **CsiSTL02** | Santa Lucia | 29.25 | 84.0 | 29.09 | M | *tongensis* |
| **CsiSTL03** | Santa Lucia | 29.97 | 85.0 | 31.38 | M | *tongensis* |
| **CsiKSB01** | Kosi Bay | 28.23 | 82.0 | 29.12 | M | *tongensis* |
| **CsiKSB02** | Kosi Bay | 26.65 | 76.0 | 26.09 | F | *tongensis* |
| **CsiKSB03** | Kosi Bay | 28.57 | 85.0 | 30.32 | M | *tongensis* |
| **CsiKSB04** | Kosi Bay | 28.31 | 85.0 | 27.88 | M | *tongensis* |
| **CsiKSB05** | Kosi Bay | 27.44 | 80.0 | 31.33 | F | *tongensis* |
| **CsiKSB07** | Kosi Bay | 29.31 | 84.0 | 30.7 | M | *tongensis* |
| **CsiKSB10** | Kosi Bay | 27.35 | 78.0 | 28.02 | F | *tongensis* |
| **CsiKSB12** | Kosi Bay | 28.07 | 80.0 | 26.02 | F | *tongensis* |
| **CsiKSB13** | Kosi Bay | 29.02 | 78.0 | 27.99 | F | *tongensis* |
| **CsiKSB16** | Kosi Bay | 27.04 | 87.0 | 27.08 | M | *tongensis* |
