# Supplementary material for: The Ecological and Geographic Context of Morphological and Genetic Divergence in an Understorey-Dwelling Bird
Source: PLoS One. 2014 Feb 7;9(2):e85903. doi: 10.1371/journal.pone.0085903 (PMC3917827; doi:10.1371/journal.pone.0085903)
Supplement: Table S3 — Effect of geography and sex on morphological variation in the brown scrub-robin as revealed by a two-way ANOVA. Nfemales = 29, Nmales = 52. (DOC) [file pone.0085903.s005.doc]

| **Trait** | **Effect** | **df** | ***F*** |
| --- | --- | --- | --- |
| Mass | geography | 11 | 14.175^***^ |
|  | sex | 1 | 8.026^**^ |
|  | geography*sex | 10 | 0.593 |
| Wing | geography | 11 | 6.669^***^ |
|  | sex | 1 | 49.072^***^ |
|  | geography*sex | 10 | 2.033 |
| Tarsus | geography | 11 | 2.033^*^ |
|  | sex | 1 | 17.690^***^ |
|  | geography*sex | 10 | 0.448 |
| Size | geography | 11 | 5.057^***^ |
|  | sex | 1 | 16.373^***^ |
|  | geography*sex | 10 | 1.694 |
