# Supplementary material for: The Ecological and Geographic Context of Morphological and Genetic Divergence in an Understorey-Dwelling Bird
Source: PLoS One. 2014 Feb 7;9(2):e85903. doi: 10.1371/journal.pone.0085903 (PMC3917827; doi:10.1371/journal.pone.0085903)
Supplement: File S1 — Multivariate approach to test niche overlap – R script. (DOC) [file pone.0085903.s006.doc]

**Multivariate approach to test niche overlap - R-Script.**

# read file where BioClim variables are stored

Bioclim <- envelope[,5:23]

# Install Vegan package

library(vegan)

# PCA using function rda

PCA <- rda(Bioclim, scale = TRUE)

biplot(PCA, scaling = 3, type = c("text", "points"))

# define the PC score for each occurrence point

PCA$x[,1] ->PC1

PCA$x[,2] ->PC2

PCA$x[,3]-> PC3

# bind the 3 vectors (PC1, PC2, PC3) into a dataframe

cbind(PC1, PC2, PC3)->PCscores3var

# Group Centroids, (Pooled) Variances

library(sda)

# use PCxcor as xx and subspecies as yy

X = as.matrix(PCscores3var[,1:3])

Y = envelope$subspecies

# determine centroids for each PC

centroids(X, Y)

### install libraries

library (caret)

library (permute)

## A randomisation test

# First, create a function to compute the diference of means for two groups along PC_i, where i is the axis of interest (PC1, PC2 and PC3)

meanDif <- function(x, grp) {

- mean(x[grp == "signata"]) - mean(x[grp == "tongensis"])}

# build null dist

Dniche1 <- numeric(length = 1000)

N <- nrow(PCscores3var)

set.seed(39)

for(i in seq_len(length(Dniche_i) - 1)) {

permute <- shuffle(N)

Dniche_i[i] <- with (PCscores3var, meanDif(PC_i,subspecies[permute]))

}

Dniche_i[1000] <- with(PCscores3var, meanDif(PC_i, subspecies))

# calculate the number of randomized values that are equal to or larger than the observed

Drand<- sum(Dniche_i >= Dniche_i[1000])

# permutational p-value

Drand/length(Dniche_i)
